# VGAEDTI: drug-target interaction prediction based on variational inference and graph autoencoder

**DOI:** 10.1186/s12859-023-05387-w

**Published:** 2023-07-06

**Authors:** Yuanyuan Zhang, Yinfei Feng, Mengjie Wu, Zengqian Deng, Shudong Wang

**Affiliations:** 1 Yinfei Feng Qingdao University of Technology, Qingdao, China; 2 School of Computer Science and Technology, China University of Petroleum, Qingdao, China

**Keywords:** Drug-target interaction prediction, Variational inference, Graph autoencoder, Variational expected maximum algorithm, Drug repurposing

## Abstract

**Motivation:**

Accurate identification of Drug-Target Interactions (DTIs) plays a crucial role in many stages of drug development and drug repurposing. (i) Traditional methods do not consider the use of multi-source data and do not consider the complex relationship between data sources. (ii) How to better mine the hidden features of drug and target space from high-dimensional data, and better solve the accuracy and robustness of the model.

**Results:**

To solve the above problems, a novel prediction model named VGAEDTI is proposed in this paper. We constructed a heterogeneous network with multiple sources of information using multiple types of drug and target dataIn order to obtain deeper features of drugs and targets, we use two different autoencoders. One is variational graph autoencoder (VGAE) which is used to infer feature representations from drug and target spaces. The second is graph autoencoder (GAE) propagating labels between known DTIs. Experimental results on two public datasets show that the prediction accuracy of VGAEDTI is better than that of six DTIs prediction methods. These results indicate that model can predict new DTIs and provide an effective tool for accelerating drug development and repurposing.

## Introduction

The therapeutic effect of a drug on a disease from its action on a target protein and its effect on its expression [1]. Therefore, the accurate identification of DTIs is of significance for understanding the treatment of disease by drugs. Recent studies have estimated the average cost of developing a new drug is around 40 million dollars, the cost of approving a drug for marketing is around 873 million dollars, and it usually takes more than a decade for a new drug to go from development to clinical use. Due to some side effects, less than 10% of new drugs have been approved for clinical medicine [2, 3]. In order to increase the number of drug approvals and reduce the cost of drug research and development, drug repurposing has attracted more and more attention from the pharmaceutical industry, namely, the use of currently approved drugs to treat new diseases [4]. For example, Gleevec, originally used to treat leukaemia, was redirected to treat gastrointestinal stromal tumours [5, 5], but the side effects of Gleevec in humans are substantial. Through making full use of drug, target and disease information, identifying DTIs play a crucial role in drug discovery, reducing the time and cost required for drug development and repurposing.

Traditional calculation methods [6] mainly include ligand-based methods [7] and molecular docking-based methods [8, 9]. For ligand-based method, the prediction accuracy is often poor because few ligands are binding to known target proteins. For molecular docking-based methods, if the 3D structure of target proteins cannot be obtained, these methods will be limited to some extent. To address the limitations of traditional methods, researchers have proposed methods to predict DTIs using machine learning which are mainly divided into two categories: (1) feature-based methods [10, 11] and (2) graph-based methods [13, 14]. Feature-based methods transform DTIs prediction into a binary classification problem and use machine learning methods such as Support Vector Machine (SVM) as classifiers [15]. For example, autoencoder-based approaches predict DTIs by maintaining consistency in pharma chemical properties and functions. Sun et al. using autoencoder to predict DTIs in the space of drug and target [16]. Zhao et al. [17] predicted drug-disease association using graph representation learning through constructing a heterogeneous network. Graph-based methods describe complex interactions between different entities, assuming that interconnected nodes tend to have more associations [18, 19]. In graph-based methods, the similarity between drugs and targets is calculated based on local or global topological information in heterogeneous graphs constructed by association information [20]. The multi-view network embedding of DTIs prediction based on consistency and complementary information preservation was constructed by Shang et al. [21]. Most of the methods currently in use, such as residual neural networks and multiscale autoencoders, learn the features of drugs and targets [22, 23], but they are shallow learning methods, which cannot fully extract the deep and complex associations between drugs and targets.

In recent years, heterogeneous networks of some deep learning algorithms have integrated information related to multiple drugs, diseases and targets for DTIs prediction. Compared with homogeneous networks, heterogeneous networks cover multiple entities and complex interaction relationships between different types of entities [24]. For example, DTINet is a method that focuses on learning the low-dimensional vector representation of drugs and targets [18], which can accurately represent the topological information of every node in the heterogeneous network. However, network-based methods focus on building various heterogeneous networks [25] but ignore the inherent feature between different types of entities. It is difficult to extract the critical feature information between nodes.

**Fig. 1:**
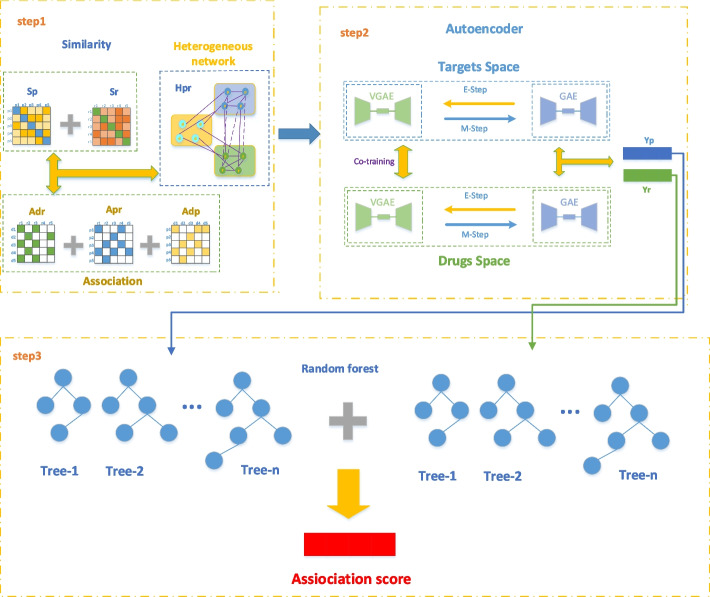
Framework of VGAEDTI. step1: the two drug and target similarity matrices obtained by similarity calculation were combined with the drug-disease, disease-target and drug-target association matrices to obtain a heterogeneous network; step2: this heterogeneous network is fed into two autoencoders to train alternately, followed by co-training, and finally the feature representation of drug and target is obtained; step3: these two features are fed into the random forest for classification

In this paper, we propose a new prediction model named VGAEDTI in Fig. 1, which combines multi-source data in a collaborative training approach to extract features of drugs and targets. We use two algorithms for feature inference and label propagation. The label propagation process may not fully utilize the low-dimensional representation learned from high-dimensional features, so under the variational inference algorithm of the Graph Markov Neural Network (GMNN) [26], the algorithm of feature inference and label propagation is integrated. Specifically, the feature inference network in VGAEDTI is designed as VGAE [27] which learns representations from the feature matrices of drugs and targets, respectively. In addition, the label propagation network in our model is GAE [28] that estimates the score of an unknown drug-target pair from known drug-target pair. These two autoencoders learn features and propagation labels alternately and are trained using a variational EM algorithm [29]. The framework minimizes the difference between the representations learned separately by the two autoencoders. In order to improve the performance of DTIs prediction, we use the Random Forest module as a classifier [30], which take the feature information of the drugs and targets obtained above as input to predict DTIs.

The major contributions of this research are as follows:

1. The VGAEDTI model uses multi-source drug information and target similarity to build a heterogeneous network, learning their embeddings through known association relationships and unknown associations.
2. The in-depth features of drugs and targets are learned through collaborative training with VGAE and GAE in VGAEDTI model.

## Materials

The two datasets we use were downloaded from several public databases, DrugBank, UniProt and MalaCard. DrugBank contains information on the molecular structure of drugs, target proteins, etc. UniProt is a protein-related database with a large amount of protein information. MalaCard is a human disease database that collects information on symptoms and related drug data. We download the chemical structure information of drugs and the targets information of all chemical drugs from DrugBank. Protein sequence information was obtained from UniProt, and drug indications were obtained from the MalaCard database. These two datasets use involves 3508 targets, 2015 drugs, 9702 diseases, and contains 207,540 known drug-disease association information and and 8947 known DTIs and some other types of data, these two data sets were summarized into Table 1.

**Table 1:** The full data for both datasets are as follows

Category	Number
Drugs	2015
Target protein	3508
Disease	9702
*Associated*	
Known drugs-diseases	207,540
Known drug-targets	8947
Drug side effects	732
Protein domain	2348

## Methods

### Drug and target similarity calculation

**Fig. 2:**
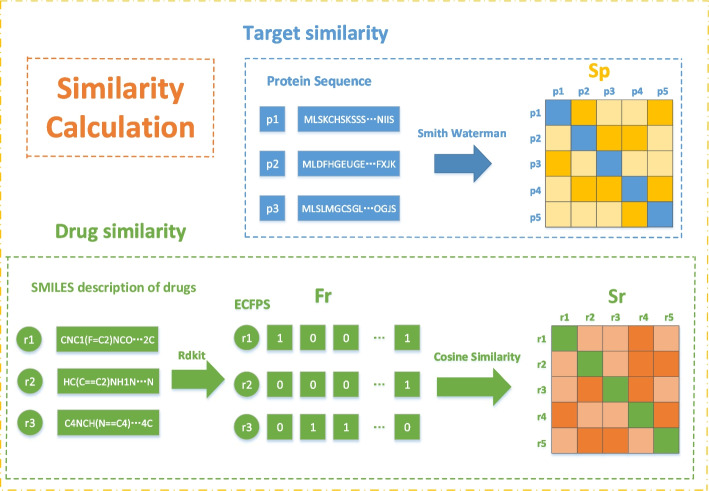
Similarity calculation diagram of drug and protein. The similarity of protein sequences was calculated using smith waterman algorithm, and the similarity of their drug smiles was calculated using cosine similarity

The n drugs in the dataset are denoted by $R=${${r}_{1}$, ${r}_{2}$, ${r}_{3}$, ……, ${r}_{n}$}, transforming SMILES structures of drug molecules into extended connectivity fingerprints (ECFPs) by using Rdkit tools, the vector of the specific structural representation of drug ${r}_{\mathrm{i}}$ is denoted by ${F}_{i}^{r}$ in Fig. 2. Cosine similarity was used to calculate the similarity between drugs and drugs as follow,

1 $$S_{r} \left( {i,j} \right) = \frac{{F_{i}^{r} \cdot F_{j}^{r} }}{{\left| {\left| {F_{i}^{r} } \right|} \right|\left\| {F_{i}^{r} } \right\|}},s_{r} \left( {i,j} \right) \in \left[ {0,1} \right] ,$$

where ${F}_{i}^{r}$ and ${F}_{j}^{r}$ in formula (1) represent the ECFPs of drug ${r}_{i}$ and drug ${r}_{j}$, respectively. The more similar the drugs are to each other, the closer the value of ${S}_{r}\left(i,j\right)$ is to 1, and a drug similarity matrix ${S}_{r}\in {R}_{n\times n}$ is obtained. Similarly, drug side effects and protein domains were calculated and fused into the drug similarity matrix and protein similarity matrix, respectively.

The $m$ targets in the dataset are denoted by $p=${${p}_{1}$, ${p}_{2}$, ${p}_{3}$, ……, ${p}_{m}$},the similarity between target protein sequence ${p}_{i}$ and target protein sequence ${p}_{j}$ can be calculated by Smith-Waterman algorithm [31], and then normalized by the following,

2 $$S_{p} \left( {i,j} \right) = \frac{{sw\left( {i,j} \right) - \min \left( {sw_{i} } \right) }}{{\max \left( {sw_{i} } \right) - \min \left( {sw_{i} } \right)}},$$

where $sw(i,j)$ in the formula (2) represents the protein similarity score calculated by Smith Waterman algorithm for two target protein sequences, $\mathrm{max }\left({sw}_{i}\right)$ and $\mathrm{min}({sw}_{i})$ represent the highest and lowest scores between protein sequence $i$ and other protein sequences, respectively, Then the target similarity matrix ${S}_{p}\in {p}_{m\times m}$ is obtained by normalization of Eq. (2).

### Construction of heterogeneous networks of drugs, targets and diseases

In order to better extract the internal connections between drug and target nodes, and perform deep learning on the common topological information representation of drug and target nodes, a heterogeneous network ${H}_{pr}$ containing drug, target and disease subnetworks is constructed, which integrates the internal connections and target similarity matrix ${S}_{p}$ and drug similarity matrix${S}_{r}$. Heterogeneous networks contain three kinds of nodes $N=${$N_{r} \; \cup \;N_{p} \; \cup \;N_{d}$} and four kinds of edges $E=${${E}_{dr}\cup {E}_{rr}\cup {E}_{pr}\cup {E}_{pp}\cup {E}_{dp}$}, If there is a known association between the drugs and the targets, there is a solid edge between them; If not, it is a dashed edge.

The adjacency matrix of a heterogeneous network of drugs, targets, and diseases is represented as follows,

3 $$H_{pr} = \left[ {\begin{array}{*{20}c} {S_{p} } & {A_{pr} } & { A_{dp} } \\ {A_{pr}^{T} } & { S_{r} } & {A_{dr} } \\ {A_{dp}^{T} } & {A_{dr}^{T} } & 0 \\ \end{array} } \right],$$

where ${S}_{r}$ belongs to drug similarity matrix, ${S}_{p}$ belongs to target similarity matrix, ${A}_{pr}$ belongs to drug target association matrix, ${A}_{dr}$ is the disease-drug association matrix and ${A}_{dp}$ is the disease-target matrix.

### Integrate drug and targets spatial information based on VGAE and GAE

VGAE and GAE serve as feature extractors for drug space and targets space. These two autoencoders extract the potential feature information from the two Spaces through feature inference and label propagation, respectively. For a drug or target node, the association and similarity with it can be regarded as the feature attribute of the node, So take *H p r* as a drug and the characteristics of the target node matrix $X$. The input to the VGAE and GAE is X. Each layer of VGAE and GAE is a graph convolutional layer. The formula for the first graph convolutional layer is as follows,

4 $$M_{encoder}^{\left( l \right)} = \sigma \left( {\tilde{D}^{{ - \frac{1}{2}}} \tilde{A}\tilde{D}^{{ - \frac{1}{2}}} X_{p}^{{\left( {l - 1} \right)}} W^{\left( l \right)} } \right).$$

For example, for the targets space, $\widetilde{A}$ is an associational adjacency matrix with self-cycle, $\widetilde{A }={A}_{pr}+{A}_{dp}+I$, $\widetilde{D}$ is the diagonal matrix of the associative adjacency matrix ${A}_{pr}+{ A}_{dp}$, $\sigma$ is the nonlinear activation function, ${X}_{p}$ is the feature matrix of target the initial input, $l$ denotes the number of layers, and ${W}^{(l)}$ denotes the weight of the $l$ layer in the network, the same is true for the drug space.

The decoding process of VAE is as follows,

$$M_{decoder}^{\left( n \right)} = \sigma { }\left( {W_{decoder}^{\left( l \right)} M_{encoder}^{\left( l \right)} + b_{decoder}^{\left( l \right)} } \right)$$

We use VGAE to extract the spatial information of the input target feature matrix ${X}_{p}$, and we can obtain the representation ${\text{ Z}}_{p}$ by the reparameterization technique as follows,

5 $$Z_{p} = \mu + \sigma \in ,$$

where $\mu { }$ represents the mean of the VGAE, $\sigma { }$ represents the standard deviation, and the random variable $\in \sim \left( {0,1} \right)$ conforms to Gaussian sampling

For the targets space, the loss function of VGAE is the sum of reconstruction error ${ }L_{VG}$ and KL divergence ${ }L_{KL}$ as follows,

6 $$L_{pVGAE} = L_{VG} + L_{KL} .$$

If the feature follows Gaussian distribution, the reconstruction error is the mean square error, when the feature follows Bernoulli distribution, the reconstruction error is cross-entropy loss as follows,

7 $$L_{{VG}} = {\text{ }}\left\{ {\begin{array}{*{20}c} {\frac{1}{2}\left\| {X_{p} - X_{p}^{\prime } } \right\|_{F}^{2} \quad if\quad X_{p} \in {\text{Gaussian}}\;{\text{distribution}}} \\ { - \sum\limits_{{ij}} {X_{p} } \log X_{p}^{\prime } \quad if\quad X_{p} \in {\text{ Bernoulli}}\;{\text{distribution}}} \\ \end{array} ,} \right.$$

where ${X}_{p}$ is the feature matrix of the input target space, ${L}_{KL}$ divergence loss can be calculated by the following equation,

8 $$L_{KL} = { } - \mathop \sum \limits_{ij} \frac{1}{2}\left( {1 + 2\log \sigma_{ij} - \mu_{ij}^{2} - \mu \sigma_{ij}^{2} } \right).$$

For the target space, the following equation is the reconstruction error ${L}_{pGAE}$ of the GAE as follow,

9 $${ }L_{pGAE} = { } - \mathop \sum \limits_{ij} A_{pr} \log A_{{pr{ }}}^{^{\prime}} ,$$

where ${A}_{pr}$ represents the input drug-target association matrix, ${A}_{pr }^{\mathrm{^{\prime}}}$ is the reconstructed drug-target matrix, and the same is true for the drug space.

We propose the VGAEDTI model, design a representation learning framework that integrates the feature inference network and labels propagation network and use the integrated variational inference to train the variational EM algorithm. VGAEDTI alternates the following two steps until convergence occurs.

E-step (Feature inference): The VGAE is used for feature inference.

M-step (Label propagation): The GAE is used for label propagation.

## Variational EM algorithm

Taking training spatial target information as an example, the variational EM algorithm is implemented by alternately minimizing the loss of the VGAE and GAE, after the variational EM algorithm alternately trains the two autoencoders until convergence as follows,

10 $$L_{{EM}} = \frac{1}{2}\left\| {Z_{p} - Z_{p}^{\prime } } \right\|_{F}^{2} ,$$

where ${Z}_{p}$ represents the output of VGAE, ${{Z}_{p}}^{\mathrm{^{\prime}}}$ represents the output of GAE, and the mean square error is used to achieve loss construction, the same is true for the drug space.

### Collaborative training integrates information from drug space and target space

In this paper, the VGAE and GAE are co-trained, and the co-training loss is represented by learning from drug and target space respectively as follows,

11 $$L_{Z} = { }\frac{1}{2}Y_{p} - X^{2}_{pF} + \frac{1}{2}Y_{r} - X^{2}_{rF}$$

In the above equation, ${Y}_{p}$ and ${Y}_{r}$ represent the protein and drug feature matrices obtained through training, where ${X}_{p}$ and ${X}_{r}$ is the initial input feature matrix, the mean square error is used to achieve loss construction.

The total optimized loss ${L}_{TVGAE}$ of the VGAE trained in target and drug space is as follows,

12 $$L_{TVGAE} = \alpha L_{pVGAE} + \left( {1 - \alpha } \right)L_{rVGAE} + \beta L_{KL} ,$$

It indicates that $\alpha$ and $\beta \in (\mathrm{0,1})$ are weight parameters to balance the information obtained from drug and target Spaces. ${L}_{pVGAE}$ belongs to the loss of target space under the VGAE and ${L}_{rVGAE}$ belongs to the loss of drug space under the VGAE.

The total optimized loss ${L}_{TGAE}$ of the GAE trained in target and drug space is as follows,

13 $$L_{TGAE} = { }\alpha L_{pGAE} + { }\left( {1 - \alpha } \right)L_{rGAE} .$$

### Prediction of DTI by random forest module

In this paper, in order to get better score prediction and avoid the negative impact of feature dimension and the importance of feature information on the prediction of drugs and targets, a Random Forest classifier [32] is used. Random Forests are a composed integrated decision tree algorithm, it belongs to integrated Bagging methods of learning [33]. By adding a random (sample randomness and properties of randomness), it can come out a high dimension data, and there is no dimension reduction, without having to make feature selection, it can judge the critical degree of the feature, and the interaction between different features. For unbalanced data sets, it can balance the error, if a large part of the features is lost, the accuracy can still be maintained. This model has strong robustness and generalization ability, so it has been widely used in the field of bioinformatics. In our learning, the learning steps of random forest are as follows,

14 $$Y = \left[ {{ }Y_{p} + { }Y_{r} { }} \right],$$

where ${Y}_{r}$ represents the feature information in the drug space and ${Y}_{p}$ represents the feature information in the target space, these two features are input into the Random Forest.

1. The first step is to sample the data. The samples in the training set are sampled in the form of put back, and the data set is sampled for $N$ times to train $N$ Classification and Regression Tree (CART) decision trees.
2. Then, the Gini coefficient is used to calculate the optimal segmentation variable, and the decision tree is constructed by node attribute splitting.
3. Obtain N decision trees by repeating the previous steps $N$ times, and predict drug target association according to the decision tree results.

The Gini coefficient is as follows,

15 $$Gini_{{index\left( {Y,f} \right)}} = { }\mathop \sum \limits_{V = 1}^{V} \frac{{\left| {Y^{V} } \right|}}{\left| Y \right|}Gini\left( {Y^{V} } \right),$$

16 $$Gini\left( {Y^{V} } \right) = 1 - \mathop \sum \limits_{i = 1}^{\left| Y \right|} U_{i}^{2} ,$$

where Y is the sample set, ${U}_{i}$ is the proportion of the $ith$ classification in Y, ${Y}^{V}$ is the sample set of Y with the $V$ value of $f$, and $f$ is the feature attribute set. We take the low-dimensional feature representations ${Y}_{p}$ and ${Y}_{r}$ obtained through autoencoder training as input. In the training stage, pairs of drugs and targets form the training set. Then put it into the Random Forest as input, and finally get the DTIs score matrix.

## Experiment and discussion

### Comparison with other methods

In order to evaluate the performance of our proposed VGAEDTI model for predicting DTIs. We use fivefold cross-validation. The dataset we use contained 1307 drugs, and the dataset was randomly divided into five groups of the same size, one of which was the test set in turn, and the remaining four groups were the training set. All the known drug target information were positive samples, and the remaining unknown drug target associations were negative samples, and the negative data contained all unknown or nonexistent DTI, it can be seen from Table 1 that imbalanced datasets were used. The VGAEDTI model was used for training. In order to better compare the superiority of our model, we also use Luo et al.’s dataset for testing and training, and our VGAEDTI model compares the following methods as follows,

GRMF: DTIs prediction using graph regularized matrix factorization [34].

DTINet: A network integration method for predicting DTIs and computing drug repurposing from heterogeneous information.

MolTrans: Transformer of molecular interactions for DTIs prediction [35].

NGDTP: Graph convolution autoencoder and Generative adversarial network approach for predicting DTIs [36].

DeepDTNet: Identify targets between known drugs by deep learning from heterogeneous networks [37].

AEFS: An autoencoder-based approach to predict DTIs by maintaining consistency in pharmacochemical properties and functions [16].

HNM: Drug repositioning by integrating target information through a heterogeneous network model [40]

The epochs of our VGAEDTI model are 500, the learning rate is 0.1, the weight decay rate is ${1e}^{-8}$, the size of the hidden layer is 256, the initial weight of the drug and protein space is 0.5, and the Adam optimizer is used to optimize.

We adopted a fivefold cross-validation method for training, and the following are some evaluation indicators:

17 $${ }Specificity = \frac{TN}{{TN + FP}} = 1 - FPR,$$

18 $$Sensitivity = \frac{TP}{{TP + FN}} = TPR = Recall,$$

19 $$Accuracy = \frac{TP + TN}{{TP + FN + TN + FP}},$$

20 $$Precision = \frac{TP}{{TP + FP}}.$$

In the above formula, TN is the true negative; FN is a false negative; FP is a false negative, TP is truly positive, FPR is the false positive rate, and TPR is the true positive rate. TPR and FPR can draw receiver operating characteristic (ROC) curves, and the area under the ROC curve (AUROC) and the area under the accuracy-recall curve (AUPR) are important indicators to measure the performance and stability of binary classification models.

### Comparison of experimental results

In order to better demonstrate that our method can extract deep drug-target information from high-dimensional feature information, In order to maintain the fairness of the experiment, we used the same data processing methods, and the input data were the same. The scores of the other models were derived from AEFS [16], we compared other six methods as follows,

Table 2 shows the comparison of AUROC and AUPR score between our VGAEDTI model and the other six methods. It can be seen intuitively that the performance of our model is superior to that of the other methods. On the first dataset, the VGAEDTI model had the best performance (AUROC = 0.9847, AUPR = 0.8247). Compared with the GRMF method, the AUROC of our method was 0.13 higher, and the AUPR was 0.61 higher. The AUROC was 0.02 higher, and the AUPR was 0.5 higher than that of AEFS, Our method is 1% higher than the AUPR of HNM. In the second dataset, the performance of the VGAEDTI model was better (AUROC = 0.9484, AUPR = 0.7302). Compared with the MolTrans method, the AUROC of our method was 0.07 higher, and the AUPR was 0.42 higher. The AUROC of our method was 0.2 lower than that of NGDTP. The AUROC was slightly higher than that of AEFS, and the AUPR was 0.31 higher, The AUPR of our method is about 13% lower than that of HNM, which may be due to the integration of our method into the omics data, leading to the better AUPR effect than our method. Our model can perform so well in the above indicators; several methods are used in front of the shallow card model, which is not good for extracting the feature attributes in the network structure, and our model uses two since the encoder, interval training, better from drug and protein extraction to better comparison, the results of the six methods in this Table 2 are derived from Sun et al. [16].

**Table 2:** AUROC and AUPR for the two datasets

Method	Dataset1		Dataset2	
	AUROC	AUPR	AUROC	AUPR
GRMF	0.8580	0.5100	0.8680	0.5300
DTINet	0.8410	0.3610	0.8850	0.2650
MolTrans	0.8890	0.0260	0.8850	0.2600
NGDTP	0.8430	0.3150	0.9690	0.1760
DeepDTNet	0.9060	0.3720	0.9060	0.3720
HNM	0.8764	0.8108	0.8831	**0.8627**
AEFS	0.9760	0.5610	0.9440	0.7160
VGAEDTI	**0.9840**	**0.8247**	**0.9485**	0.7302

Bold numbers indicate the highest scores

**Fig. 3:**
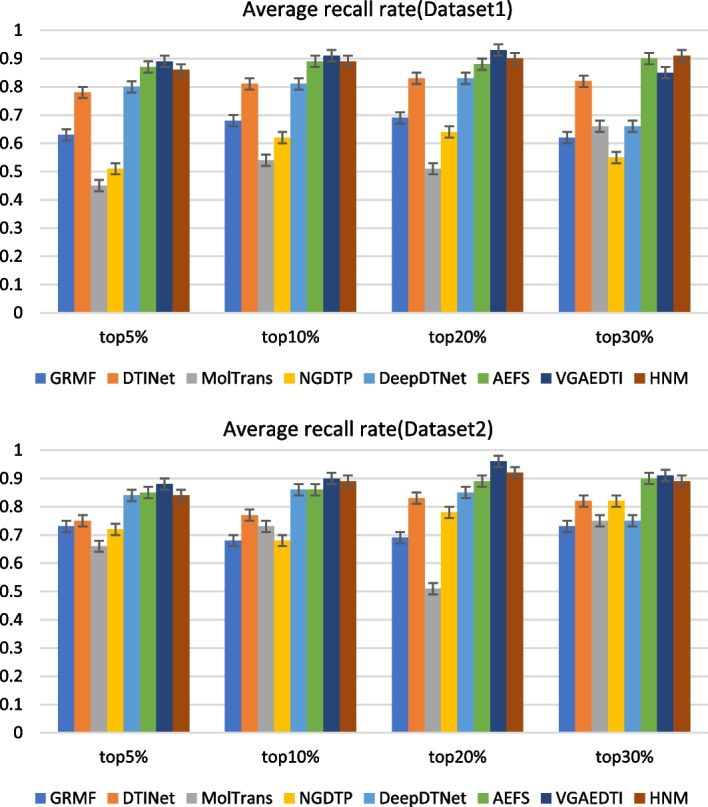
Average recall rates of 8 methods under two datasets

In order to better evaluate the performance of the model, we decided to use the recall rate of the top k DTIs candidates (5%, 10%, 20%, 30%). The recall rate can reflect whether the model can reasonably predict the performance of DTIs. We still selected the average recall rate of these methods to compare the performance of these methods with our method, as shown in Fig. 3.

In the first data set, the average value of recall of our model before (5%, 10%, 20%) is better than that of the six methods, and in the first 30%, our method is slightly lower than AEFS. In the second dataset, our model outperformed all DTIs methods in the top (5%, 10%, 20%, 30%), reflecting our model's strong performance in identifying drug-target associations.

## Case study

Evaluating the performance of a model is mainly based on accuracy and practicality. We trained the VGAEDTI model using known DTIs datasets to predict the natural association of drug-targets. We will predict the interaction between drug-target scores in the top 15 for recording. In order to verify the accuracy of the prediction score, we verified its authenticity by querying the source data set of Uniprot and DrugBank databases; the database contains a large number of drugs and targets of the associated information, so that supported by data authenticity.

In the Table 3, these target associations were confirmed in both Uniprot and DrugBank databases, at the same time, we found that drugs DB00007 (Leprolide) and DB00014 (Goserelin) in the Table 3 have effects on prostate disease [38], and drug DB00007 is associated with target protein P30968 Gonadotropin-releasing hormone receptor). Drug DB00007 and target protein P22888 (Lutropin-choriogonadotropic hormone receptor) ranked high in the scores of our model results, so they have a unknown association. If this association can be predicted, it could have important implications for the discovery of new treatments for diseases. In order to have a better visual understanding of the interaction between proteins and molecules, such as P30968 and DB00007, they are two interacting drug-target pairs. Pharmaceutical chemists need to understand the role of targets in the human body or pathogens in the process of disease, so as to design drugs that can regulate the physiological functions of targets, so as to achieve the purpose of treating diseases. A drug may have multiple potential targets in the body at the same time. When a drug acts on its target, it is called on-target, and it acts on other targets, it is called Off-Target. In general, a disease may be associated with multiple targets, and a target may be associated with multiple diseases. How to identify and select the key targets is very important for drug design. Our VGAEDTI model can screen a large number of unknown but related drug targets in advance, reduce the blind test of drug targets for researchers, save the cost of some unnecessary biological experiments, and shorten the time of drug development and promote the pace of drug research and development.

**Table 3:** Top 15 drug-target interaction pairs

Rank	Drug ID	Protein ID	Evidence	Rank	Drug ID	Protein ID	Evidence
1	DB00007	P30968	UniProt, DrugBank	9	DB00996	P54687	Uniprot
2	DB00014	P22888	DrugBank	10	DB00999	P55017	Uniprot, DrugBank
3	DB00314	P9WJ63	Uniprot, DrugBank	11	DB01403	P08912	Uniprot, DrugBank
4	DB00718	P24024	Uniprot, DrugBank	12	DB04839	P07288	Uniprot, DrugBank
5	DB00774	P43166	Uniprot, DrugBank	13	DB06694	P25100	Uniprot, DrugBank
6	DB00798	P98164	Uniprot	14	DB08868	O95977	Uniprot, DrugBank
7	DB00834	P07288	Uniprot, DrugBank	15	DB11596	P0A827	Uniprot, DrugBank
8	DB00918	P28221	Uniprot, DrugBank				

**Fig. 4:**
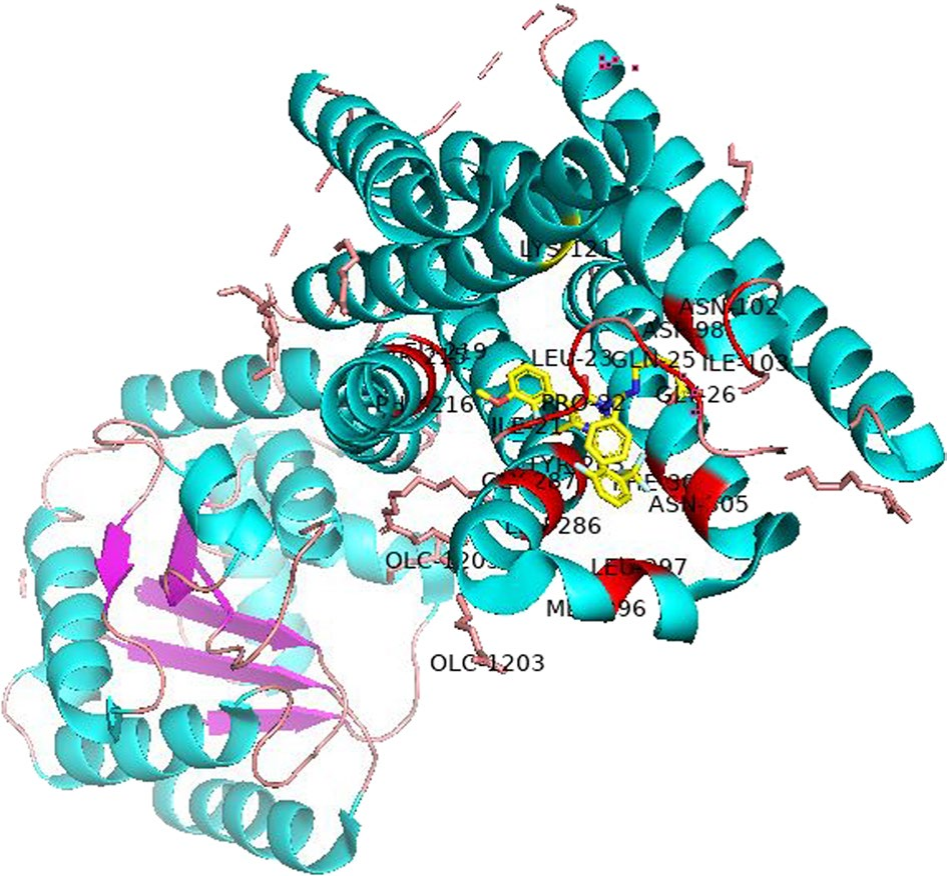
DTI pairs predicted by VGAEDTI

To further validate this novel interaction, we performed computational docking and utilized the docking program AutoDock to infer the possible binding modes of the new predicted DTI. Docking results showed that Gentamicin can dock the structure of 2M0P. More specifically, Ibrutinib binds to 2MOP by forming hydrogen bonds with residues LEU-23, PBU-22, and ASN-305.We use pymol for molecular docking and hydrogen bond coloring, as shown in Fig. 4.

### Ablation experiment

VGAEDTI model combines drugs space and target space information, so two spatial information are integrated to co-training improve the ability of its important feature information extraction. The pattern of co-training on performance evaluation of the VGAEDTI model has an important influence. Therefore, this paper set up a set of ablation experiments on its effectiveness.

**Fig. 5:**
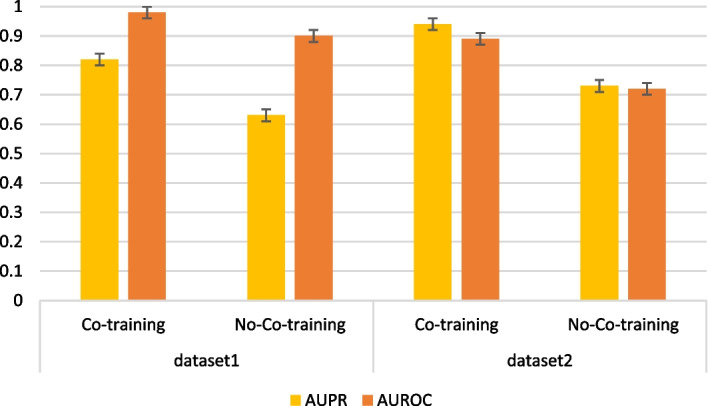
AUROC and AUPR with and without co-training in two datasets

The AUROC and AUPR of the VGAEDTI model with and without co-training under two different datasets are shown in Fig. 5. Except for these two Settings, all other parameters are consistent to ensure the accuracy of the experiment. In dataset 1, the AUROC score was 0.98 with a co-training and 0.90 without co-training, while the AUPR was 0.82 and 0.63, respectively. In dataset 2, the AUROC score for using co-training is 0.89, the AUPR score for not using co-training is 0.72, and the AUPR score is 0.94 and 0.73, respectively. The above two datasets show that the prediction performance of the model using co-training is higher than that of the model not using co-training. Therefore, the experimental results show that the VGAEDTI model can extract the feature information of drug space and target space to predict DTIs accurately, and co-training is essential.

**Fig. 6:**
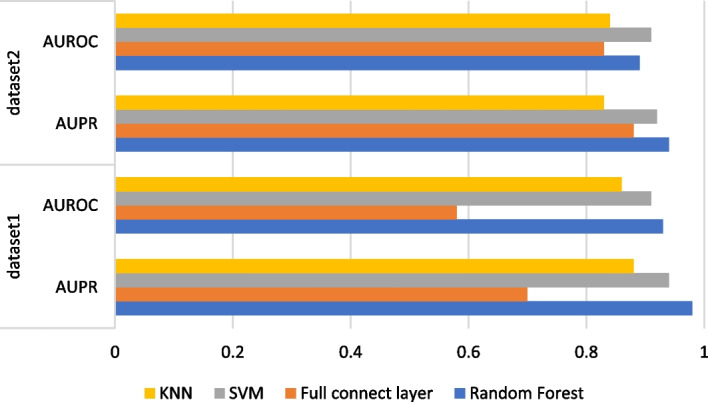
Comparison of the performance of different classifiers on two datasets

In VGAEDTI, based on the embedding features of drug and target, we use random forest to calculate drug-target association scores. In order to confirm that random forest can obtain better score prediction, we performed the following ablation experiments as Fig. 6. We used several different classifiers, fully connected layer, SVM, KNN as well as random forest to compare the performance of the two datasets. In the first dataset, random forest (AUPR = 0.98, AUROC = 0.93) and SVM (AUPR = 0.94,AUROC = 0.91) were used, and random forest performed better than other classifiers in this dataset. In the second dataset, The AUROC of random forest is 2% higher than that of SVM, but it is still superior to other classifiers. It can be seen that the importance of the Random Forest classification module for this VGAEDTI model enhances the accuracy of the scoring results.

### Weight parameter selection of drug space and target space

By integrating the feature information of drug space and target space trained alternately by two autoencoders using a variational EM algorithm, the VGAEDTI model can get more accurate feature information so as to better predict its association. To select the suitable weight parameters of the two spaces to maintain the balance between them and ensure the contribution of different spatial feature information outputs to the prediction performance of the model, we use different datasets for testing.

**Fig. 7:**
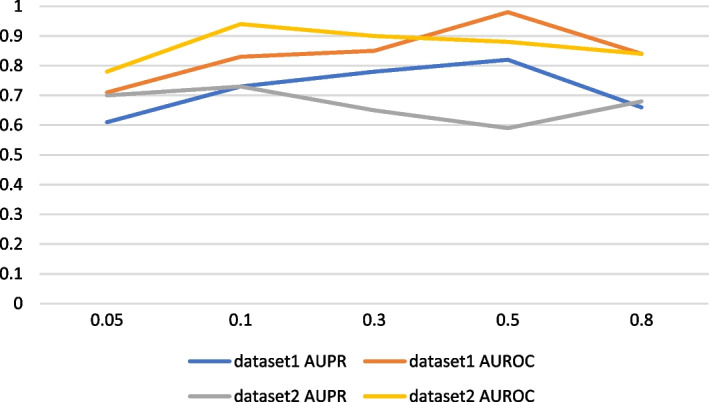
AUROC and AUPR for different weights in drug and target Spaces in two datasets

It can be seen from the above Fig. 7 that when our VGAEDTI model integrates spatial feature information of drugs and proteins, it can be seen in dataset 1 that when the weight is 0.5, AUROC is 0.98, and AUPR is 0.82. The prediction performance of the model at this time is the best. In dataset 2, when the weight is 0.1, AUROC is 0.94, AUPR is 0.73, and the prediction performance for dataset 2 is the best. Experiments can show that different data sets contribute different weights to the feature information of integrated drug and target space, and some properties, such as the sparsity of data sets, affect the model’s training.

## Conclusion

How to accurately identify DTIs is one of the most important steps in drug repurposing and new drug development. In this study, we propose a novel model VGAEDTI to predict DTIs. Firstly, the VGAEDTI model calculates the similarity of multi-source drug information, target information and disease information, and then constructs a heterogeneous network through the known association information and feature information among them, so as to better extract more potentially complex relationships among drugs, targets and diseases. Then it is input to the VGAE and GAE for feature information extraction. The VGAE deduces the feature representation from the drug and target space respectively, while the GAE propagates the label between the known drug and target associations, and uses the variational EM algorithm for alternating training until convergence. Also, the co-training starategy is used to capture the feature information of drug space and target space, which enhances the ability of VGAEDTI to capture efficient low-dimensional representations from high-dimensional features, thereby improving the robustness and accuracy of predicting the unknown DTIs. In this way, the obtained drug and target feature information is more accurate and comprehensive. In order to obtain better score prediction and avoid the negative effects of feature dimension and importance of feature information on predicting drugs and targets, we use random forest classifier, which can judge the importance of features and the interaction between different features. For imbalanced data sets, it can balance the error. If a large part of the features are lost, the accuracy can still be maintained, and the model has strong robustness and generalization ability. In order to evaluate the performance of the proposed VGAEDTI model for predicting DTIs. We use fivefold cross validation to compare the performance of six methods on two different datasets, all of which achieved better results in some aspects, and also proved that our model has strong generalization ability. In general, our model VGAEDTI can be used as an effective and accurate tool for predicting DTIs.

## Future and prospects

Although VGAEDTI model has good performance at present, there are also some potential drawbacks in extracting information from heterogeneous networks, recently inspired by Zhao et al. [39], existing computational models can only use low-level biological information at the level of individual drugs, diseases and targets and their associations. This also germinates new ideas for the next work, in the future work, not only multi-source information but also high-order meta-path information of heterogeneous networks should be integrated to improve the prediction performance and generalization performance of the model.

## Data Availability

All instructions and codes for our experiments are available at https://github.com/FengYinFei/VGAEDTI.
